# Deciphering the contributions of fecal microbiota from patients with high-grade glioma to tumor development in a humanized microbiome mouse model of glioma

**DOI:** 10.1093/noajnl/vdaf085

**Published:** 2025-04-25

**Authors:** Cheng Wang, Yiqi Fan, Lu Zhang, Zhanyi Zhao, Feiyang Luo, Kaijian Sun, Meiqin Zeng, Hao Tian, Meichang Peng, Yunhao Luo, Hailin Zhao, Shuai He, Haitao Sun

**Affiliations:** Neurosurgery Center, The National Key Clinical Specialty, The Engineering Technology Research Center of Education Ministry of China on Diagnosis and Treatment of Cerebrovascular Disease, Guangdong Provincial Key Laboratory on Brain Function Repair and Regeneration, The Neurosurgery Institute of Guangdong Province, Zhujiang Hospital Institute for Brain Science and Intelligence, Zhujiang Hospital, Southern Medical University, Guangzhou 510280, China; Clinical Biobank Center, Microbiome Medicine Center, Guangdong Provincial Clinical Research Center for Laboratory Medicine, Department of Laboratory Medicine, Zhujiang Hospital, Southern Medical University, Guangzhou 510280, China; Department of Pharmacy, Zhujiang Hospital, Southern Medical University, Guangzhou 510280, China; Neurosurgery Center, The National Key Clinical Specialty, The Engineering Technology Research Center of Education Ministry of China on Diagnosis and Treatment of Cerebrovascular Disease, Guangdong Provincial Key Laboratory on Brain Function Repair and Regeneration, The Neurosurgery Institute of Guangdong Province, Zhujiang Hospital Institute for Brain Science and Intelligence, Zhujiang Hospital, Southern Medical University, Guangzhou 510280, China; Clinical Biobank Center, Microbiome Medicine Center, Guangdong Provincial Clinical Research Center for Laboratory Medicine, Department of Laboratory Medicine, Zhujiang Hospital, Southern Medical University, Guangzhou 510280, China; Neurosurgery Center, The National Key Clinical Specialty, The Engineering Technology Research Center of Education Ministry of China on Diagnosis and Treatment of Cerebrovascular Disease, Guangdong Provincial Key Laboratory on Brain Function Repair and Regeneration, The Neurosurgery Institute of Guangdong Province, Zhujiang Hospital Institute for Brain Science and Intelligence, Zhujiang Hospital, Southern Medical University, Guangzhou 510280, China; Clinical Biobank Center, Microbiome Medicine Center, Guangdong Provincial Clinical Research Center for Laboratory Medicine, Department of Laboratory Medicine, Zhujiang Hospital, Southern Medical University, Guangzhou 510280, China; Neurosurgery Center, The National Key Clinical Specialty, The Engineering Technology Research Center of Education Ministry of China on Diagnosis and Treatment of Cerebrovascular Disease, Guangdong Provincial Key Laboratory on Brain Function Repair and Regeneration, The Neurosurgery Institute of Guangdong Province, Zhujiang Hospital Institute for Brain Science and Intelligence, Zhujiang Hospital, Southern Medical University, Guangzhou 510280, China; Clinical Biobank Center, Microbiome Medicine Center, Guangdong Provincial Clinical Research Center for Laboratory Medicine, Department of Laboratory Medicine, Zhujiang Hospital, Southern Medical University, Guangzhou 510280, China; Neurosurgery Center, The National Key Clinical Specialty, The Engineering Technology Research Center of Education Ministry of China on Diagnosis and Treatment of Cerebrovascular Disease, Guangdong Provincial Key Laboratory on Brain Function Repair and Regeneration, The Neurosurgery Institute of Guangdong Province, Zhujiang Hospital Institute for Brain Science and Intelligence, Zhujiang Hospital, Southern Medical University, Guangzhou 510280, China; Neurosurgery Center, The National Key Clinical Specialty, The Engineering Technology Research Center of Education Ministry of China on Diagnosis and Treatment of Cerebrovascular Disease, Guangdong Provincial Key Laboratory on Brain Function Repair and Regeneration, The Neurosurgery Institute of Guangdong Province, Zhujiang Hospital Institute for Brain Science and Intelligence, Zhujiang Hospital, Southern Medical University, Guangzhou 510280, China; Clinical Biobank Center, Microbiome Medicine Center, Guangdong Provincial Clinical Research Center for Laboratory Medicine, Department of Laboratory Medicine, Zhujiang Hospital, Southern Medical University, Guangzhou 510280, China; Neurosurgery Center, The National Key Clinical Specialty, The Engineering Technology Research Center of Education Ministry of China on Diagnosis and Treatment of Cerebrovascular Disease, Guangdong Provincial Key Laboratory on Brain Function Repair and Regeneration, The Neurosurgery Institute of Guangdong Province, Zhujiang Hospital Institute for Brain Science and Intelligence, Zhujiang Hospital, Southern Medical University, Guangzhou 510280, China; Clinical Biobank Center, Microbiome Medicine Center, Guangdong Provincial Clinical Research Center for Laboratory Medicine, Department of Laboratory Medicine, Zhujiang Hospital, Southern Medical University, Guangzhou 510280, China; Neurosurgery Center, The National Key Clinical Specialty, The Engineering Technology Research Center of Education Ministry of China on Diagnosis and Treatment of Cerebrovascular Disease, Guangdong Provincial Key Laboratory on Brain Function Repair and Regeneration, The Neurosurgery Institute of Guangdong Province, Zhujiang Hospital Institute for Brain Science and Intelligence, Zhujiang Hospital, Southern Medical University, Guangzhou 510280, China; Clinical Biobank Center, Microbiome Medicine Center, Guangdong Provincial Clinical Research Center for Laboratory Medicine, Department of Laboratory Medicine, Zhujiang Hospital, Southern Medical University, Guangzhou 510280, China; Neurosurgery Center, The National Key Clinical Specialty, The Engineering Technology Research Center of Education Ministry of China on Diagnosis and Treatment of Cerebrovascular Disease, Guangdong Provincial Key Laboratory on Brain Function Repair and Regeneration, The Neurosurgery Institute of Guangdong Province, Zhujiang Hospital Institute for Brain Science and Intelligence, Zhujiang Hospital, Southern Medical University, Guangzhou 510280, China; Clinical Biobank Center, Microbiome Medicine Center, Guangdong Provincial Clinical Research Center for Laboratory Medicine, Department of Laboratory Medicine, Zhujiang Hospital, Southern Medical University, Guangzhou 510280, China; Department of Neurosurgery, Guangzhou Institute of Cancer Researsh, the Affiliated Cancer Hospital, Guangzhou Medical University, Guangzhou, China; Department of Pharmacy, Zhujiang Hospital, Southern Medical University, Guangzhou 510280, China; Key Laboratory of Mental Health of the Ministry of Education, Guangdong–Hong Kong–Macao Greater Bay Area Center for Brain Science and Brain-Inspired Intelligence, Southern Medical University, Guangzhou 510280, China; Neurosurgery Center, The National Key Clinical Specialty, The Engineering Technology Research Center of Education Ministry of China on Diagnosis and Treatment of Cerebrovascular Disease, Guangdong Provincial Key Laboratory on Brain Function Repair and Regeneration, The Neurosurgery Institute of Guangdong Province, Zhujiang Hospital Institute for Brain Science and Intelligence, Zhujiang Hospital, Southern Medical University, Guangzhou 510280, China; Clinical Biobank Center, Microbiome Medicine Center, Guangdong Provincial Clinical Research Center for Laboratory Medicine, Department of Laboratory Medicine, Zhujiang Hospital, Southern Medical University, Guangzhou 510280, China

**Keywords:** glioma, gut microbiota, *Merdimonas*, metabolite, *s*phingosine 1-phosphate

## Abstract

**Background:**

Recent studies have revealed associations between gut microbiota and glioma. However, the underlying mechanisms remain poorly understood. This study primarily aims to elucidate the impact of altered gut microbiota on tumor progression in glioma-bearing mice.

**Methods:**

Fecal samples were collected from glioma patients and healthy controls to compare the effects of human-derived gut microbiota on glioma development in mice. We also analyzed the associations between these microbiota profiles and plasma metabolites.

**Results:**

Significant differences were observed in both the composition and diversity of the gut microbiota between glioma patients and healthy controls. Mice transplanted with gut microbiota from high-grade glioma patients (HGG-FMT) exhibited accelerated glioma progression compared to those transplanted with microbiota from healthy individuals (HC-FMT). Specifically, *Eisenbergiella*, *Mailhella*, and *Merdimonas* were significantly enriched in HGG-FMT mice, while *Limosilactobacillus* and *Anaerospora* increased in HC-FMT mice. Furthermore, *Merdimonas* showed a positive correlation with sphingosine, sphingosine 1-phosphate, and D-sphingosine in HGG-FMT mice. Conversely, *Limosilactobacillus* was positively correlated with stearidonic acid and eicosapentaenoic acid in HC-FMT mice.

**Conclusions:**

Our findings demonstrate that gut microbiota from high-grade glioma patients can promote glioma progression in mice, potentially through mechanisms involving sphingosine 1-phosphate. This metabolite may enter the bloodstream and accelerate glioma growth, offering novel insights into glioma pathogenesis and potential treatment options.

## Graphical Abstract

**Graphical Abstract:**
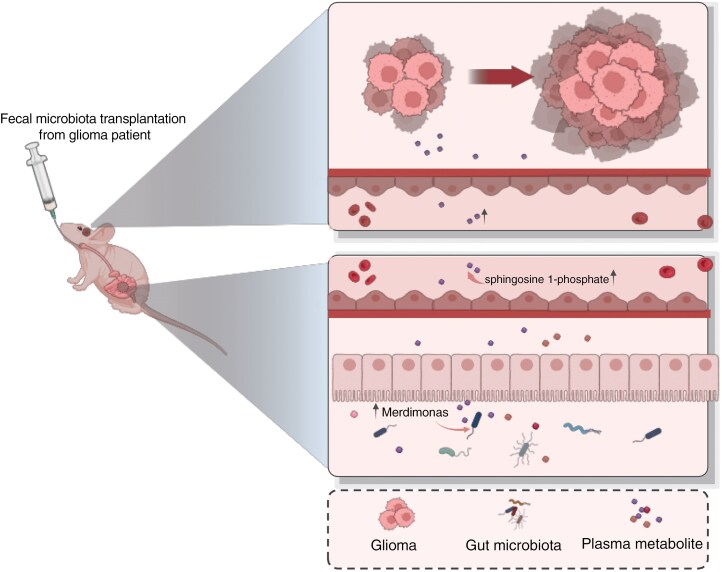

**Key Points**

- Gut microbes differ between glioma patients and healthy people.
- Transplanting fecal microbiota from high-grade glioma patients promotes glioma progression in mice.
- Gut microbiota may regulate glioma growth through metabolites.

**Importance of the Study**

Glioma is the most prevalent primary malignant brain tumor in adults, with clinical outcomes remaining suboptimal. Despite ongoing research into targeted therapies and immunotherapies, significant improvements in patient prognosis have yet to be achieved. Clinical studies have demonstrated an association between alterations in gut microbiota and glioma development, while basic research has confirmed that alterations of gut microbiome can influence glioma progression. However, these studies often lack systematic and comprehensive design, and the underlying mechanisms remain poorly understood.

This study integrates clinical and basic research to systematically investigate the effects of human gut microbiota on glioma growth. In addition, we explore the potential role of microbiota-regulated metabolites in glioma progression, offering new insights for the development of novel therapeutic strategies for glioma.

Glioma is the most prevalent primary malignant tumor of the central nervous system (CNS). Despite aggressive treatment strategies aimed at reducing tumor burden, such as surgical resection and chemotherapy, the intrinsic invasive nature and immune evasion capabilities of gliomas result in high recurrence rates and poor prognoses.^1–3^ The rapid proliferation and invasive growth patterns of gliomas, coupled with molecular heterogeneity and the blood-brain barrier complicating drug delivery, limited the effectiveness of therapeutic interventions.^4^ Despite advances in basic and clinical glioma research, improvements in overall survival rates remain elusive.^1,5^ This underscores the urgent need for innovative diagnostic methods and personalized therapeutic strategies.

In recent years, increasing attention has been focused on the influence of extracranial factors on the glioma microenvironment, particularly the gut microbiota, which has emerged as a significant area of interest. Studies have shown that the gut microbiome composition in glioma patients differs markedly from that of healthy controls (HCs).^6,7^ Moreover, disruptions in gut microbial homeostasis can affect the responses of glioma model mice to treatments such as temozolomide and anti-PD-L1 therapies.^8,9^ Although previous studies have indicated a regulatory role of the gut microbiota in glioma progression within mouse models, the specific mechanisms through which the gut microbiota influences glioma progression remain to be fully elucidated.^10–13^ Recent insights into the role of microbiota-derived metabolites, such as short-chain fatty acids (SCFAs), highlight their potential regulatory effects. SCFAs not only inhibit inflammatory responses but also boost antioxidant activities in pathological samples from colon and breast cancers.^14,15^ Moreover, SCFAs modulate the NF-κB pathway in cancer and immune cells, potentially enhancing glioma invasiveness.^16^ In addition, the progression of advanced glioma has been linked to lithocholic acid, a secondary bile acid metabolite produced by gut microbiota.^17^ These findings suggest that microbiota-derived metabolites play critical roles in both the development and treatment of glioma.

Although the association between gut microbiota and glioma has been established, the precise mechanisms remain unclear. In this study, we conducted fecal microbiota transplantation from humans to a mouse model of glioma and performed a correlation analysis between gut microbiota and plasma metabolites. Our objective is to validate the impact of gut microbiota on glioma progression and to explore whether this effect is mediated through metabolites.

## Materials and Methods

### Enrollment of Clinical Study Cohorts and Fecal Sample Acquisition

This nested case-control study included 52 participants, who were recruited from the Department of Neurosurgery at Zhujiang Hospital of Southern Medical University between April 2022 and July 2023. Ethical approval was obtained from the Ethics Committee of Zhujiang Hospital (approval number: 2022-KY-102-02), and the study was registered with the Chinese Clinical Trial Registry (registration number: ChiCTR2300067789). Detailed descriptions of the methods, demographic characteristics, and inclusion and exclusion criteria are provided in the **Supplementary Materials**.

### Mouse Source and Model Construction

Six-week-old female BALB/c-nude mice, provided by the Experimental Animal Center of Zhujiang Hospital, Southern Medical University, were used in this study with ethical approval granted (LAEC-2022-163).

The mice were subjected to an antibiotic regimen (ABX), receiving drinking water supplemented with ampicillin, vancomycin, neomycin, and metronidazole to prepare for subsequent procedures. Following ABX treatment, the mice were randomly assigned to 3 groups for fecal microbiota transplantation (FMT) to reconstruct their intestinal ecosystems: high-grade glioma patients (HGG-FMT), low-grade glioma patients (LGG-FMT), and healthy controls (HC-FMT).

FMT involved the collection and transplantation of fecal samples from these populations. After intestinal ecosystem reconstruction, the mice underwent intracranial implantation with U87-Luc glioma cells derived from humans. Tumor growth was monitored using in vivo bioluminescence imaging. At the end of the monitoring period (12 days), the mice were euthanized, and tissues were harvested, labeled, and stored at −80 °C for further analysis. Comprehensive details on the study design, ABX and FMT procedures, glioma cell implantation, sample collection, storage, and imaging techniques are available in the **Supplementary Materials**.

### 16S rDNA Amplicon Sequencing

#### DNA extraction and amplicon sequencing.—

All stool samples were thawed from storage at −80 °C and handled in a biosafety cabinet. Total genomic DNA was extracted using the CTAB/SDS method. The concentration and purity of DNA were assessed on a 1% agarose gel.

The V3–V4 regions of 16 S rRNA were amplified by PCR using specific primers. All PCR reactions were carried out with 15 μL of Phusion® High-Fidelity PCR Master Mix (New England Biolabs). Then, the mixture of PCR products was purified with a Qiagen Gel Extraction Kit (Qiagen, Germany). Sequencing libraries were generated using TruSeq® DNA PCR-Free Sample Preparation Kit (Illumina, USA) and assessed on the Qubit@2.0 Fluorometer (Thermo Scientific) and Agilent Bioanalyzer 2100 system. Finally, the library was sequenced on an Illumina NovaSeq platform and 250 bp paired-end reads were generated.

#### Data analysis.—

The procedures for paired-end reads handling, data splitting, sequence assembly,^18^ data filtration,^19,20^ and chimera removal^21,22^ have been detailed in previous publications. The downstream amplicon bioinformatic analyses were performed with EasyAmplicon v1.12^23^ and the procedures of the analyses were described previously.^24^ Alpha-diversity differences between groups were assessed using the Wilcoxon test. Community differences were evaluated using permutational multivariate analysis of variance (PERMANOVA), based on the Bray–Curtis distance matrix. Differences in bacterial abundances across various taxonomic levels were analyzed using linear discriminant analysis (LDA) of effect size (LEfSe).

### Untargeted Metabolomics

#### Metabolite extraction, sample preprocessing, and annotation.—

Untargeted metabolic profiling was performed using plasma samples from mice. After vortexing, homogenization, and sonication, the samples were incubated and centrifuged, and the supernatant was used for further experiments. Quality control was maintained by mixing equal aliquots of each sample’s supernatant. For analysis, 10 µL of the mixture was injected into an ultra-high-performance liquid chromatography (UHPLC) system (Vanquish, Thermo Fisher Scientific, USA) coupled with a mass spectrometer (MS) (Orbitrap Elite, Thermo). The chromatography parameters were optimized, including column specifications, temperature settings, and a binary mobile phase, with linear gradient elution. To prevent carryover effects, a blank injection was performed following each sample injection.

Positive and negative mode MS data were acquired using the default data-dependent acquisition method. Metabolite data preprocessing included conversion to mzML format, processed with an in-house program which was developed using the R and based on XCMS, for peak detection, extraction, alignment, and integration. Then an in-house MS2 database (BiotreeDB) was applied to metabolite annotation. The cutoff for annotation was set at 0.3. Compounds were functionally and taxonomically annotated using the Kyoto Encyclopedia of Genes and Genomes and Human Metabolome databases.

#### Analysis of metabolite profiles.—

Quality control (QC) samples were created by pooling equal fractions of each sample and were processed alongside the experimental samples to ensure analytical consistency. A QC sample was injected after every 10 samples throughout the analytical sequence to assess the stability of the untargeted metabolomics workflow. Data normalization was performed through area normalization. An Orthogonal Partial Least Squares Discriminant Analysis (OPLS-DA) model was used, incorporating Variable Importance in Projection (VIP) scores and Student’s *t*-test *P* values (analyzed using SPSS 20.0) to identify significantly differing features between groups. Features with a VIP score greater than 2 and a *P*-value less than .05 were selected for metabolite annotation. Metabolite identification was conducted using MetDNA^25^ and a self-built database, with annotations sourced from the Human Metabolome Database (HMDB) (https://hmdb.ca/) and the Kyoto Encyclopedia of Genes and Genomes (KEGG) (https://www.kegg.jp/).

### Data Analysis

Data were analyzed using GraphPad Prism 8.0 software (GraphPad Software Inc, La Jolla, CA, USA). Results are expressed as mean ± standard deviation. Spearman’s correlation analysis, conducted using SPSS 20.0, was employed to examine the relationships between bacterial levels and metabolite intensities. Differences between the two groups were assessed using independent, unpaired two-tailed Student’s *t*-tests. For non-normally distributed data, the Wilcoxon rank-sum test was utilized, unless specified otherwise.

## Results

### Alterations of Gut Microbiota in Human Cohorts

**Figure 1.**
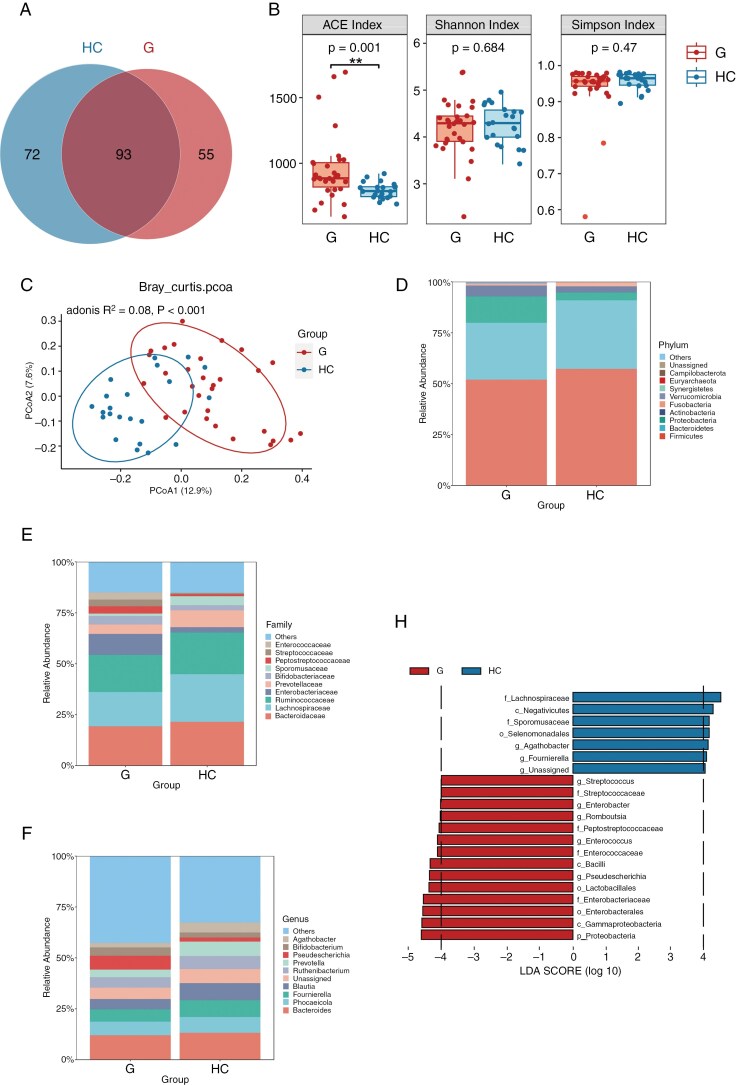
Differences in fecal microbiota between glioma (G) group and healthy control (HC) group based on 16S rRNA sequencing analysis. (A) Venn diagram illustrating the number of Amplicon sequence variants (ASVs). (B) Employing ACE, Shannon, and Simpson indices to assess microbial α-diversity. (C) β diversity analysis represented by principal coordinates analysis (PCoA) plot based on Bray–Curtis, demonstrating the distinct clustering of microbial composition between groups with Adonis test. (D–F) Taxonomic distributions of gut microbiota from G and HC groups at phylum, family, and genus level. (H) Histogram of linear discriminant analysis (LDA) score showing the discriminative taxa based on linear discriminant analysis effect size (LEfSe) analysis with LDA score > 4.0 between G and HC groups.

We analyzed the gut microbiota of glioma patients and healthy individuals using 16S rDNA amplicon sequencing. Amplicon sequence variants revealed both common and unique microbiota in the glioma (G) and HC groups (**Figure 1A**). To estimate species diversity within these groups, we utilized alpha-diversity indices including ACE, Simpson, and Shannon. A notable increase in the ACE index among glioma patients compared to HCs suggests a greater number of microbiome species in the former group (**Figure 1B**). Principal coordinate analysis (PCoA), based on the Bray–Curtis distance matrix and analyzed through PERMANOVA, demonstrated significant differences in microbial composition between the two groups (*Adonis*, *P* < .001; **Figure 1C**).

The variations in gut microbiota at the phylum, family, and genus levels are illustrated in **Figure 1D–F**. LEfSe analysis revealed statistically significant disparities in relative abundance (*P* < .05, LDA > 4.0). At the family level, *Enterobacteriaceae*, *Enterococcaceae*, *Peptostreptococcaceae*, and *Streptococcaceae* were significantly increased in glioma patients, while *Lachnospiraceae* and *Sporomusaceae* were predominantly found in HCs. At the genus level, *Pseudescherichia*, *Enterococcus*, *Enterobacter*, and *Streptococcus* were markedly enriched in glioma patients, whereas *Agathobacter* and *Fournierella* were significantly more abundant in HCs (**Figure 1H**).

### Gut Microbial Alteration and Glioma Progression of Mice after Fecal Microbiota Transplantation Derived from Human

The differences in gut microbiota between glioma patients and HCs suggested a potential impact of gut microbiota on glioma progression. To further investigate the result, we reconstructed the intestinal microecology of mice using human fecal microbial suspensions to assess the effect of altered gut microbiota on glioma progression. 16S amplicon sequencing was employed to monitor changes in intestinal microbial characteristics during the FMT process in three mouse models. The successful establishment of mouse models harboring three types of human-derived gut microbiota was confirmed (Supplementary Figure 3).

**Figure 2.**
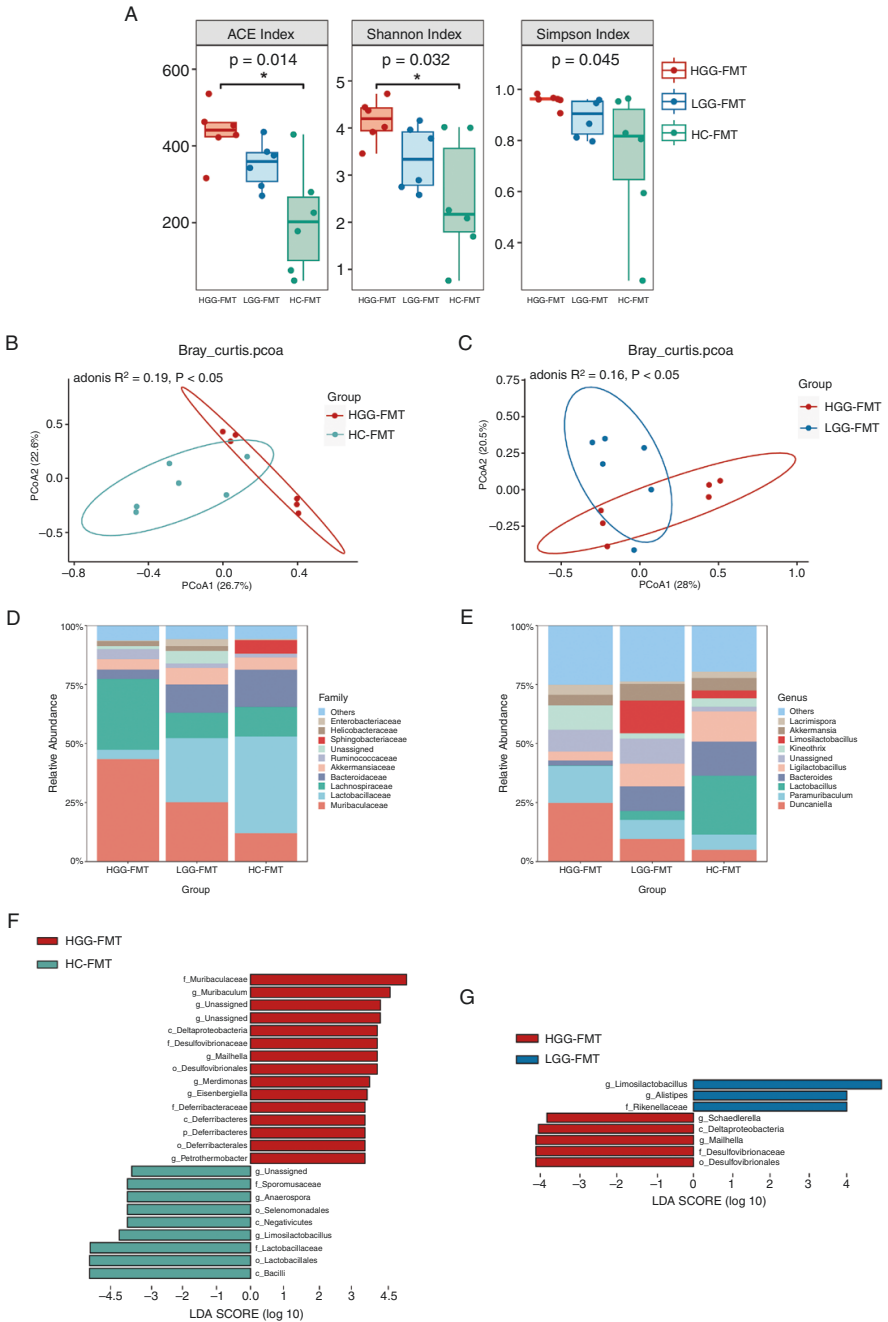
Gut microbiota composition in mice after fecal microbiota transplantation from patients with high-grade glioma and low-grade glioma, and healthy controls. (A) Employing ACE, Shannon, and Simpson indices to assess microbial α-diversity among groups. (B and C) β diversity analysis represented by PCoA plot based on Bray–Curtis, demonstrating the distinct clustering of microbial composition between groups with Adonis test. (D and E) Taxonomic distributions of gut microbiota among groups at family and genus levels. (F) Histogram of LDA score showing the discriminative taxa based on LEfSe analysis with LDA score > 4.0 between HGG-FMT and HC-FMT groups. (G) Histogram of LDA score showing the discriminative taxa based on LEfSe analysis with LDA score > 3.0 between HGG-FMT and LGG-FMT groups.

Following FMT, we observed higher microbiome richness and evenness in HGG-FMT compared to HC-FMT, with significant differences noted in the ACE and Shannon indices (**Figure 2A**). Principal coordinate analysis (PCoA) and PERMANOVA revealed significant differences in intestinal microbial composition among the groups (*Adonis*, *P* < .05; **Figure 2B and C**).

At the taxonomic level, variations in gut microbiota at the family and genus levels among the groups were depicted (**Figure 2D and E**). LEfSe analysis (*P* < .05, LDA > 3.0) revealed that *Muribaculaceae*, *Desulfovibrionaceae*, *Deferribacteraceae*, *Mailhella*, *Merdimonas*, *Eisenbergiella*, and *Petrothermobacter* were significantly enriched in the HGG-FMT, while *Lactobacillaceae*, *Sporomusaceae*, *Limosilactobacillus*, and *Anaerospora* were significantly increased in the HC-FMT (**Figure 2F**). Compared to LGG-FMT, *Desulfovibrionaceae*, *Mailhella*, and *Schaedlerella* were significantly enriched in the HGG-FMT, while *Rikenellaceae*, *Limosilactobacillus*, and *Alistipes* were significantly decreased (**Figure 2G**).

**Figure 3.**
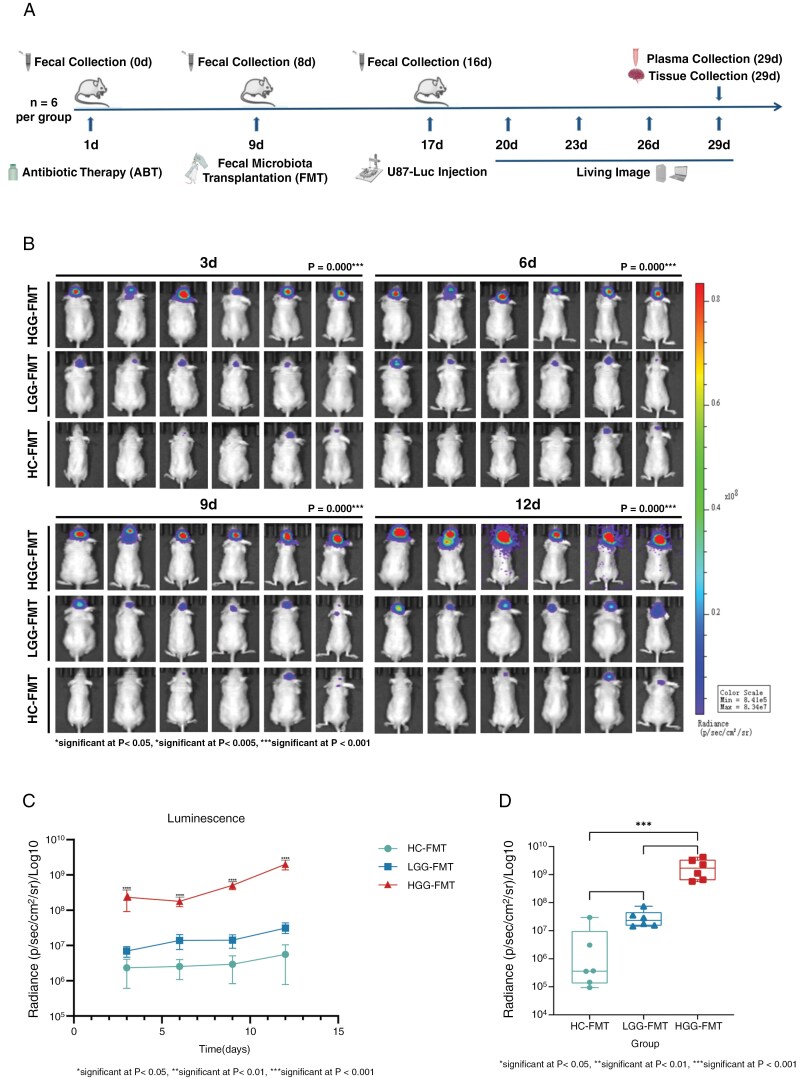
Overview of the impact of fecal microbiota transplantation (FMT) on tumor growth in a mouse model. (A) Schematic representation of the experimental timeline outlining fecal collection at 0, 8, and 16 days, antibiotic therapy, FMT, U87-Luciferase glioma cell injection, and subsequent bioluminescence imaging and sample collection endpoints. (B) Bioluminescence imaging showing the progression of glioma growth in live mice at 3rd, 6th, 9th, and 12th day post-injection of U87-Luc glioma cells. The images are categorized into three groups based on the source of FMT. (C) Quantitative analysis of luminescence intensity over time, comparing tumor growth dynamics among the HGG-FMT, LGG-FMT, and HC-FMT groups. (D) Boxplot distribution of luminescence intensity on 12th day post U87-Luc injection, comparing the final tumor burden among HGG-FMT, LGG-FMT, and HC-FMT groups.

Tumor progression was monitored on 3, 6, 9, and 12 days after intracranial injection of tumor cells in mice (**Figure 3A and B**). The results demonstrated that mice in HGG-FMT exhibited more significant tumor progression compared to LGG-FMT and HC-FMT (*P* < .001) (**Figure 3C**). At the terminal observation period (12 days post-tumor implantation), statistical analyses revealed that the tumor progression in the HGG-FMT was significantly faster than HC-FMT (*P* < .001), with no significant differences observed between the LGG-FMT and either the HGG-FMT or HC-FMT (**Figure 3D**).

These findings suggested that the gut microbiota from patients with high-grade glioma can promote tumor progression in mice compared to that from HCs, indicating that gut microbiota alteration can modulate glioma progression.

### Plasma Metabolite Variations in Mice

**Figure 4.**
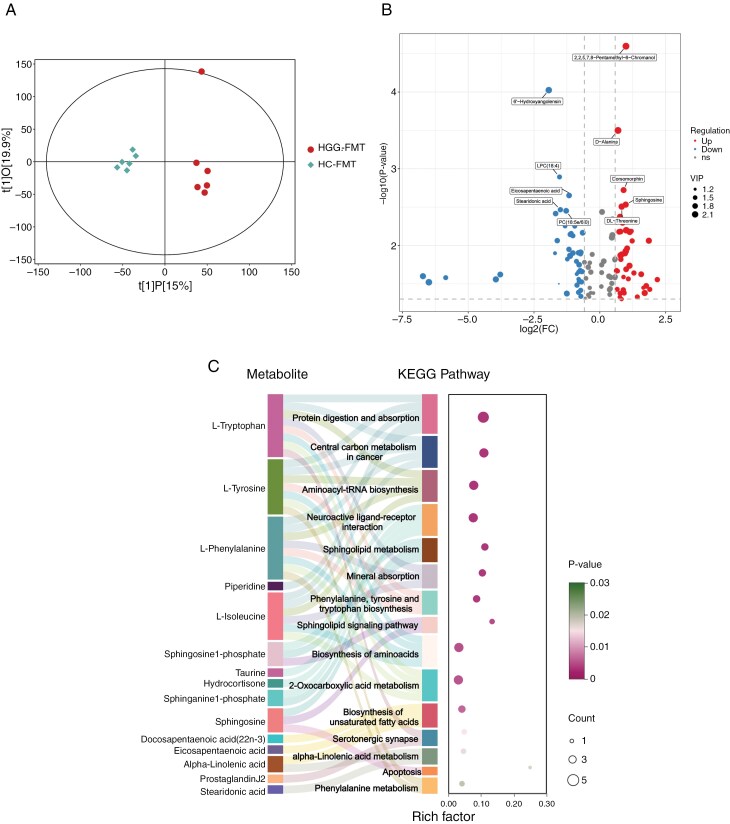
Mouse plasma metabolomics analysis between HGG-FMT and HC-FMT groups. (A) OPLS-DA score plots of plasma metabolites from mouse models between HGG-FMT and HC-FMT groups. (B) Volcano plots of differential plasma metabolites with log2FC > 1.5, VIP > 2, and *P* < .01, indicating the differences in the expression of metabolites in HGG-FMT compared to HC-FMT group. (C) KEGG pathway enrichment analysis of the top 15 differential plasma metabolites between HGG-FMT and HC-FMT groups. The color of bubbles represents the value of *P* value (Fisher’s test), bubble size corresponds to the count of differential metabolites in each pathway, abscissa value represents the ratio of the number of annotated differential metabolites in a pathway to the number of all metabolites in that pathway.

To investigate whether gut microbiota from humans influences glioma growth through plasma metabolites, we conducted an untargeted metabolomic analysis of plasma from HGG-FMT and HC-FMT glioma mice. The results identified 1577 metabolites with significant differences between groups. Analysis using an OPLS-DA model showed a clear separation of all plasma metabolites between the HGG-FMT and HC-FMT (**Figure 4A**), with the model’s effectiveness validated by the OPLS-DA permutation test (Supplementary Figure 4A and B).

Secondary mass spectrometry qualitative analysis generated a clustering heatmap that depicted the differentially abundant metabolites between groups (**Supplementary** Figure 4C). Metabolites with VIP score greater than 2.0 from the OPLS-DA model’s first principal component and a significance threshold of *P* < .05. A volcano plot highlighted the top 10 significantly different metabolites, revealing increases in 2,2,5,7,8-pentamethyl-6-chromanol, D-alanine, and sphingosine in HGG-FMT mice. Conversely, increases in 6’-hydroxyangolensin, LPC (18:4), eicosapentaenoic acid, stearidonic acid, and PC (18:5e/6:0) were observed in HC-FMT mice (**Figure 4B**).

**Figure 5.**
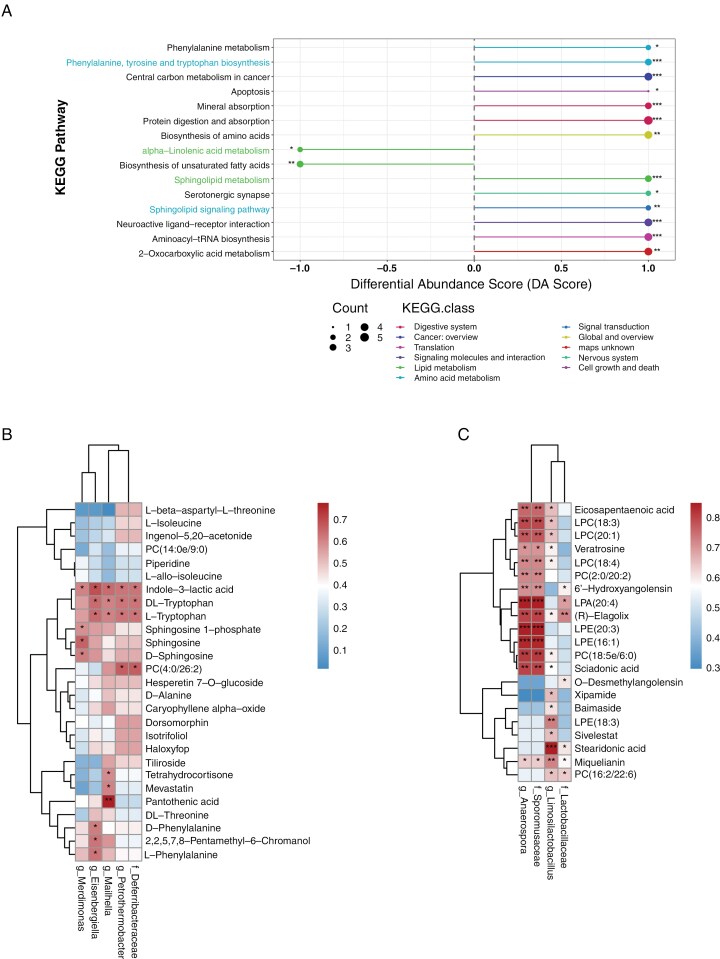
Pathway enrichment analysis and correlation analysis of gut microbiota and plasma metabolites. (A) Differential abundance score (DA score) plot for KEGG pathways in HGG-FMT compared to HC-FMT group, DA score of 1 indicates that all annotated differential metabolites in the pathway are upregulated, −1 indicates that all annotated differential metabolites in the pathway are downregulated. Bubble size corresponds to the count of differential metabolites in each pathway. Heatmap showing the Spearman correlation analysis between upregulated differential metabolites and discriminative microbial taxa in HGG-FMT group (B) and HC-FMT group (C).

Further analysis of the enrichment of differentially abundant metabolites in KEGG metabolic pathways revealed significant disruptions in sphingolipid metabolism, phenylalanine, tyrosine, and tryptophan biosynthesis, as well as the sphingolipid signaling pathway (**Figure 4C**). Differential abundance score analysis indicated that pathways such as phenylalanine, tyrosine, and tryptophan biosynthesis, aminoacyl-tRNA biosynthesis, and sphingolipid metabolism were predominantly upregulated in HGG-FMT. In contrast, the biosynthesis of unsaturated fatty acids and alpha-linolenic acid metabolism were more active in HC-FMT (**Figure 5A**).

### Correlation Analysis between Gut Microbiota and Metabolites in Mice

Spearman correlation analysis was performed to elucidate the relationships between differentially enriched gut microbiota and plasma metabolites in the HGG-FMT and HC-FMT groups. In the HGG-FMT group, a significant positive correlation was observed between differentially enriched microbes and significantly upregulated metabolites. *Eisenbergiella, Mailhella*, *Petrothermobacter*, and *Deferribacteraceae* showed strong positive correlations with DL-tryptophan and L-tryptophan. *Merdimonas* was positively correlated with sphingosine, sphingosine 1-phosphate (S1P), and D-sphingosine. *Eisenbergiella* also demonstrated positive correlations with L-phenylalanine, 2,2,5,7,8-pentamethyl-6-chromanol, and D-phenylalanine. *Mailhella* showed positive correlations with tetrahydrocortisone and mevastatin, while it had a notable correlation with pantothenic acid. In addition, *Petrothermobacter* and *Deferribacteraceae* were positively correlated with PC (4:0/26:2). Interestingly, all the differentially abundant gut microbiota were positively correlated with indole-3-lactic acid (**Figure 5B**).

In the HC-FMT group (**Figure 5C**), *Limosilactobacillus* showed positive correlations with miquelianin, LPE (18:3), baimaside, and notably with stearidonic acid. *Lactobacillaceae* was closely associated with (R)-elagolix. In addition, *Anaerospora* and *Sporomusaceae* exhibited significant positive correlations with numerous metabolites, especially LPA (20:4), LPE (20:3), LPE (16:1), sciadonic acid, PC (18:5e/6:0), and (R)-elagolix. These findings suggest that the differential gut microbiota in the HC-FMT group may be involved in regulating glioma growth through lipid metabolism processes, which warrants further investigation.

## Discussion

Glioma is the most prevalent and lethal malignant tumor within the CNS.^26^ Numerous studies have highlighted the dysregulation of gut microbial homeostasis in glioma patients,^6,7,27^ and Mendelian randomization has been employed to confirm the causal relationship between relevant microbial communities and glioma,^28^ focusing primarily on analyzing gut bacteriome alteration. However, limited research has addressed the specific relationship between gut microbiota and glioma.^9,29,30^ In this study, we confirmed the discordance of gut microbiota between glioma patients and HCs. Furthermore, we demonstrated that alterations in gut microbiota can modulate glioma progression in mice. In addition, we explored the relationships between gut microbiota and host plasma metabolites, underscoring the potential influence of gut microbiota on glioma development.

We profiled the microbiome in fecal samples from 30 glioma patients and 22 healthy individuals. In alignment with prior studies,^6,7^ significant differences in gut microbiota community diversity and structure were observed between glioma patients and HCs. Notably, we observed a significant increase in conditional pathogens in glioma patients, such as *Enterobacter*, *Enterococcus*, and *Streptococcus*. Previous studies^6,7,31^ have suggested that gut microbiota could serve as biomarkers for glioma. Our receiver operating characteristic (ROC) analysis of differential bacterial genera between the groups yielded area under the curve (AUC) values above 0.8 (Supplementary Figure 1A–F), indicating the potential of the gut microbiota as predictive biomarkers for glioma.

Previous research^27,30,32^ on gut microbiota and glioma has shown that glioma growth is associated with alterations in gut microbiota, but they have not determined whether changes in gut microbiota or glioma itself are the initiating factors in this process. Scholars^29^ have demonstrated that disruption of gut microbiota following antibiotic treatment can modulate immune homeostasis in the brain, thereby promoting cancer progression. Furthermore, an intervention study of glioma involving the intestinal transfer of cecal contents in mice has been initiated^17^, and the transplantation of *Bifidobacterium lactis* combined with *Lactobacillus* plantarum in mice was shown to reduce glioma volume and prolong survival time.^33^ These studies have primarily utilized murine-derived glioma cells to establish models, which do not fully replicate the tumor characteristics of glioma patients. Therefore, the translational applicability of these studies is limited, although they provided operational guidance and a theoretical basis for subsequent research. In our study, we employed human-derived glioma cells for intracranial transplantation to more accurately mirror the disease characteristics of human glioma. Compared to the study conducted by Wang et al.,^33^ our intervention, which involved the comprehensive transfer of fecal microbiota from both patients and HCs, aimed to reconstruct a patient-compatible intestinal microecosystem. This approach enables a more precise exploration of the influence of the gut microbiota on glioma development. We observed significantly accelerated tumor growth in the HGG-FMT compared to the HC-FMT (*P* < .001), indicating that gut microbiota from high-grade glioma patients promotes glioma progression. These findings underscore the potential of gut microbiota alterations to influence glioma progression and suggest that further investigation into gut microbiota as a treatment measure for glioma is warranted.

Correlation analysis between differentially increased gut microbiota and plasma metabolites in HGG-FMT revealed that *Merdimona*s exhibited a significant association with sphingosine, S1P, and D-sphingosine. *Merdimonas*, a strictly anaerobic genus isolated from human feces^34^ and is Gram-stain positive and considered a potential risk factor for glioma in our study. It has been shown to be increased in female depression patients^35^ and has been rarely reported in association with other diseases, making further investigation into the causal relationship between *Merdimonas* and glioma clinically significant. Sphingolipids are highly enriched in the nervous system and are crucial mediators of immune function.^36^ Intestinal microbiota harboring serine palmitoyltransferase which is essential for sphingolipid synthesis, can produce sphingolipids, which are then transferred to host epithelial tissues and hepatic portal veins.^37,38^ Sphingosine can be phosphorylated by sphingosine kinase-1 to form S1P, and S1P was closely related to cell proliferation and tumor-promoting effects.^39^ It has been reported that reduced levels of S1P in melanoma can prolong tumor survival by regulating T cell differentiation.^40^ Another study has demonstrated that GBM can trigger sphingolipid metabolism reprogramming, adaptively elevating the level of S1P to enhance invasiveness, which can form an internalization effect and isolate T cells in the bone marrow, preventing them from interacting tumor microenvironment.^41^ In our study, sphingolipid metabolism was found to be upregulated in the HGG-FMT, and sphingosine and S1P levels were also elevated in plasma. These findings suggest that *Merdimonas* may be involved in regulating S1P levels in plasma, thereby promoting glioma development.

Previous research has demonstrated that *Limosilobacillus* can ferment flaxseed, resulting in increased concentrations of flaxseed oil, which is primarily composed of α-linolenic acid.^42^ α-linolenic acids possess specific neuroprotective effects, and the conversion between eicosapentaenic acid and stearidonic acid can reduce Aβ-induced neuronal damage, oxidative stress, and inflammation.^43^ Furthermore, docosahexaenoic acid, derived from α-linolenic acid, significantly inhibits breast cancer growth and reduces tumor microvessel density.^44,45^ Combined with the upregulation of α-Linolenic acid metabolism and the correlation analysis in HC-FMT, we speculate that *Limosilobacillus* may be involved in regulating α-linolenic acid levels in plasma, potentially suppressing glioma development.

Several limitations warrant consideration for future research. To begin with, our clinical samples were derived from glioma of varying grades. Since different grades of glioma present distinct clinical manifestations and pathogenic mechanisms, we are unable to precisely describe the gut microbiota's impact on a specific type of glioma. In future research, we plan to collect clinical samples from patients with specific grade glioma to achieve more specific results. In addition, during animal model construction, there was a high mortality rate. We will refine the modeling method and adjust cell injection volumes in the future. Meanwhile, to enhance result reliability, we will increase biological replicates within groups to further verify the gut microbiota's promoting effect on glioma. Besides, we did not examine plasma metabolomics in human cohorts to cross-validate the findings from our mouse experiments. While previous studies have highlighted disruptions in the gut microbiota of glioma patients and characterized the metabolic landscape of tumor tissues,^46–48^ research into the impact of metabolic changes induced by alterations in gut microbiota remains limited. In addition, the results derived from joint analyses of animal experimental data provide only preliminary insights into potential mechanisms, necessitating further validation through more comprehensive basic research. Furthermore, this study mainly focused on the influence of gut microbiota on glioma growth. We did not track the survival time of mice and collect prognostic information from glioma patients, such as median survival time, Karnofsky Performance Status (KPS) score, and Glasgow Coma Scale (GCS) score. In the future, we will also refine the study design and incorporate more diverse tumor detection methods, such as ex vivo tumor volume measurement, MRI, or CT imaging, to provide a more comprehensive assessment of tumor progression. Analyzing the correlation between survival prognosis information and characteristic gut microbiota is a highly significant task, which deserves further investigation through large-scale clinical trials. In addition, 16S rDNA sequencing may fail to fully capture the functional or dynamic changes in the microbiome during gut microbiota transplantation. Multi-omics analytical approaches can better assess the functional relevance of microbial communities in disease progression.

In conclusion, our study establishes a link between glioma development and gut microbiota. We find that gut microbiota from high-grade glioma patients can promote glioma progression, with the underlying mechanisms potentially mediated by metabolites through the plasma pathway, as indicated by our correlational analysis. Notably, we hypothesize that *Merdimonas* may produce S1P, which could be absorbed into the bloodstream to accelerate glioma growth. This insight provides a compelling rationale for testing these strategies in clinical settings for glioma patients.

## Supplementary Material

Supplementary material is available online at *Neuro-Oncology Advances* (https://academic.oup.com/noa).

vdaf085_suppl_Supplementary_Materials

## Data Availability

The raw 16S amplicon sequence data reported in this article have been deposited in the Genome Sequence Archive in National Genomics Data Center, China National Center for Bioinformation / Beijing Institute of Genomics, Chinese Academy of Sciences (https://ngdc.cncb.ac.cn/gsa-human/: accession no.subHRA011142 and subHRA011144). The raw plasma metabolites data reported in this article have been deposited in the OMIX, China National Center for Bioinformation / Beijing Institute of Genomics, Chinese Academy of Sciences (https://ngdc.cncb.ac.cn/omix: accession no.OMIX007553). All other data supporting the findings of this study are available within the article, the Supplementary Information, or from the corresponding authors upon reasonable request.
