# Supplementary material for: Deciphering the contributions of fecal microbiota from patients with high-grade glioma to tumor development in a humanized microbiome mouse model of glioma
Source: Neurooncol Adv. 2025 Apr 25;7(1):vdaf085. doi: 10.1093/noajnl/vdaf085 (PMC12187996; doi:10.1093/noajnl/vdaf085)
Supplement: vdaf085_suppl_Supplementary_Materials [file vdaf085_suppl_supplementary_materials.docx]

**1. Inclusion and exclusion criteria for study participants**

**1.1 Inclusion criteria for glioma** **disease group**

1) The patients were diagnosed with glioma by pathological diagnosis after the first surgery;

2) The patients had no history of radiotherapy or chemotherapy before the surgery;

3) The patients had complete clinical information and essential laboratory data;

4) The patients had no history of inflammatory bowel diseases or acute/chronic gastrointestinal disease;

5) The patients had no other severe physical or mental disorders;

6) The patients or their guardian/legal representative voluntarily participated in the study and subscribed the Informed Consent Form.

**1.2 Inclusion criteria for** **healthy control group**

1) All participants were matched for age, sex, and body mass index (BMI) with the glioma disease group;

2) All participants' brain computerized tomography did not find primary or metastatic tumor in brain;

3) All participants had no history of inflammatory bowel disease or acute/chronic gastrointestinal disease;

4) All participants were not associated with other severe physical or mental disorders;

5) All participants or their guardian/legal representative voluntarily participated in the study and subscribed the Informed Consent Form;

**1.3 Exclusion criteria**

1) All participants had received antibiotics or probiotics treatment within 2 months before the biological specimens were collected or had known active bacterial, fungal, or viral infections;

2) All participants had severe systemic diseases diagnosed during the health screening: hypertension(diastolic pressure≥100 mmHg or systolic pressure≥160 mmHg), cardiovascular disease, diabetes mellitus, rheumatic disease, active phase of acute or chronic infectious diseases;

3) All participants were in pregnancy or lactation;

4) All participants had a history of cancer;

5) All participants had a history of gastrointestinal surgery or had intestinal invasive examinations within 3 months.

Glioma grading was according to the 2016 WHO classification criteria for central nervous system tumors, categorizing participants into three groups: 1) High-grade glioma group (n=15, WHO grades III-IV); 2) Low-grade glioma group (n=15, WHO grades I-II); and 3) Healthy controls (HCs, n=24). Fecal samples from glioma patients were collected preoperatively, whereas samples from the healthy control group were obtained from volunteers. All samples were stored at -80°C in the Clinical Biobank Resource Center of Zhujiang Hospital following collection.

**2. Methods for mouse model construction**

**2.1 Cell Culture**

The U87-Luc glioblastoma cells were obtained from laboratory culture and were generously provided by the Department of Neurosurgery at Zhujiang Hospital, Southern Medical University, following multiple passages. The cells were cultivated in high-glucose DMEM supplemented with 10% FBS, 100 U/ml penicillin, and 100 mg/mL streptomycin. The culture was maintained in an incubator at 37°C under a 5% CO_2_.

**2.2 Construction of a humanized gut microbiome mouse model of glioma**

Six-week-old female BABL/C-nude mice (n=18), weighing approximately 20 ± 2 grams, were used in this study. These SPF (Specific Pathogen Free) animals were provided by the Experimental Animal Center of Zhujiang Hospital, Southern Medical University, and were housed in the barrier area of the center.

After one week of acclimatization from five weeks of age, fecal samples were collected from the mice into sterile EP tubes, labeled, and stored at -80°C for subsequent analysis. The mice underwent antibiotics treatment (ABX), which involved administering drinking water supplemented with ampicillin (0.5 mg/mL), vancomycin (0.25 mg/mL), neomycin (0.5 mg/mL), and metronidazole (0.5 mg/mL) for seven consecutive days to deplete the intestinal microbiota. Following ABX, fecal samples were again collected and stored as before, and the mice were provided with fresh sterile drinking water to allow the intestinal microbiota to recover for 24 hours.

The mice were then randomly divided into three groups: HGG-FMT, LGG-FMT, and HC-FMT groups, for Fecal Microbiota Transplantation (FMT) to reconstruct the intestinal microecosystem. FMT involved collecting fecal samples from different populations (high-grade glioma patients, low-grade glioma patients, and healthy individuals) and storing them at -80°C. On the day of transplantation, the samples were thawed at 37°C, dissolved in a specific volume of saline to prepare a 400 mg/mL FMT suspension, and transplanted within 6 hours. Before introducing the bacteria, the mice were fasted for 30 minutes and given a 100 μL 1M NaHCO3 solution to buffer stomach pH. Subsequently, the HGG-FMT group received a high-grade glioma patient’s fecal suspension, the LGG-FMT group received a low-grade glioma patient’s fecal suspension, and the HC-FMT group received a healthy individual’s fecal suspension, with each mouse receiving 80-120 μL daily for seven days.

After FMT, the intestinal flora was allowed to stabilize for 24 hours, and fecal samples were collected and stored at -80°C. Twenty-four hours post-FMT, the mice underwent tumor implantation, with U87-Luc glioblastoma cells implanted into the brain, and tumor growth was monitored using in vivo bioluminescence imaging. At the experimental endpoint of tumor growth (12th day post-implantation), the mice were euthanized, and fecal, serum, and brain tissue samples were collected, labeled, and stored at -80°C for further analysis.

**2.3 Bioluminescence imaging technology**

In vivo bioluminescence imaging was conducted to monitor tumor growth on days 3, 6, 9, and 12 post-tumor inoculation. Fifteen minutes prior to imaging, the mice were intraperitoneally injected with a working solution of D-Luciferin potassium salt (AAT Bioquest®, Inc. 12507 D-Luciferin, potassium salt UltraPure Grade). Following anesthesia using the XGI-8 gas anesthesia system, imaging was performed using the IVIS® Spectrum Imaging System. The images were then analyzed with Living Image® Software 4.4 for IVIS® Spectrum.

**3. Demographic Characteristics of human cohorts**

**Table 1** provides a detailed overview of the demographic characteristics of the participants. The average age of patients with high-grade glioma (HGG) was 30 years old, while patients with low-grade glioma (LGG) had an average age of 33 years old. The average age of the healthy control group (HC) was 53 years old. The proportion of females in the high-grade glioma patient group was 66.6%, in the low-grade glioma patient group was 60%, and in the healthy control group was 54.5%. The body mass index (BMI) of patients with high-grade glioma was 23.1764 ± 2.2 kg/m^2^, and for patients with low-grade glioma, it was 22.3493 ± 2.54 kg/m^2^.

| **Table1** Demographic and clinical features of the study population in this study | | | | | |
| --- | --- | --- | --- | --- | --- |
| Characteristics | Glioma grade | | HCs(N=22) | HGG vs LGG | Glioma vs HCs |
|  | HGG(WHOⅢ/Ⅳ, N=15) | LGG(WHOⅠ/Ⅱ, N=15) |  | P | P |
| Age (years) |  |  |  | 0.695 | 0.661 |
| Range | 3-70 | 4-64 | 24-72 |  |  |
| Mean ± SD | 30.1333 ± 23.75 | 33.2667 ± 19.38 | 53.7727 ± 13.51 |  |  |
| Gender |  |  |  | 0.99 | 0.576 |
| Gender (female) | 10/15(66.6%) | 9/15(60%) | 12(54.5%) |  |  |
| BMI (kg/m2) |  |  |  | 0.354 |  |
| Range | 19.47 - 26.79 | 17.3 - 26.17 | NA |  |  |
| Mean ± SD | 23.1764 ± 2.25 | 22.3493 ± 2.54 | NA |  |  |
| ALT (U/L) |  |  |  | 0.283 |  |
| Range | 6-31 | 6-219 | NA |  |  |
| Mean ± SD | 15.8667 ± 7.79 | 31 ± 52.91 | NA |  |  |
| Blood glucose (mmol/L) |  |  |  | 0.711 |  |
| Range | 3.75-13.12 | 3.92-9.2 | NA |  |  |
| Mean ± SD | 5.3 ± 2.2 | 5.1 ± 1.2 | NA |  |  |
| Size_min_(mm) |  |  |  | 0.570 |  |
| Range | 14-52 | 20-66 | NA |  |  |
| Mean ± SD | 39.5 ± 10.0 | 35.2 ± 13.8 | NA |  |  |
| For the difference comparison of clinical characteristics between the groups, the Student’s *t*-test was employed in in cases of continuous normally distributed data. Categorical variables were compared by the χ2 test. HCs, healthy controls; LGG, low-grade glioma; HGG, high-grade glioma. | | | | | |
